# Supplementary material for: Optimal transport reveals dynamic gene regulatory networks via gene velocity estimation
Source: bioRxiv. 2024 Sep 16:2024.09.12.612590. Preprint. [Version 1] doi: 10.1101/2024.09.12.612590 (PMC11429941; doi:10.1101/2024.09.12.612590)
Supplement: Supplement 1 [file NIHPP2024.09.12.612590v1-supplement-1.pdf]

bioRxiv preprint doi: https://doi.org/10.1101/2024.09.12.612590; this version posted September 16, 2024. The copyright holder for this preprint (which was not certified by peer review) is the author/funder, who has granted bioRxiv a license to display the preprint in perpetuity. It is made available under aCC-BY 4.0 International license.

Supporting information

S1 Fig. Related to Figure 2. Other performance metrics except AUPRC on datasets simulated from HARISSA [34], including Area under Receiver Operating Characteristic (ROC) curves and early precision as defined by [22].

S2 Fig. Related to Figure 3. AUROC and EP ratio on datasets simulated from curated networks and BoolODE [22]. The EP ratio equals the early precision value divided by the random baseline, and the value should be greater than one if the performance is better than a random classifier.

S3 Fig. Dependence of median AUPRC on hyperparameters (1) (α, ϵ) for OTVelo-Corr, and (2)(λ, r) for OTVelo-Granger on HARISSA simulations. Brighter is better.

S4 Fig. Dependence of median AUPRC-based metrics on hyperparameters (1) (α, ϵ) for OTVelo-Corr, and (2)(λ, r) for OTVelo-Granger on Curated models. Brighter color is better.

S5 Fig. AUPRC given signed prediction when cross validation is performed to find (r, λ). The approach with default parameters $r = 0.5, \lambda = 1$ is labeled 'OTVelo-Granger', and 'OTVelo-CV' indicates a strategy of picking (r, λ) via a 5-fold cross validation over a grid $r \in \{0, 0.5, 1.0\}$ and $\lambda \in \{0.1, 0.4, 0.7, 1.0, 1.3, 1.6\}$, the same grid as in Figures S3 and S4.

S6 Fig. Dependence of velocity field on (α, ϵ) illustrated on one instance of FN8 dataset simulated by HARISSA. Top: velocity field with $\alpha = 0.5$ and different ϵ. Lower values of ϵ gives velocity fields of good smoothness but can take significant computational time or even fail to converge when $\epsilon < 0.001$, while bigger value of ϵ results in non-smooth velocity field. Bottom: velocity field with fixed $\epsilon = 0.01$ but different α. $\alpha = 0$ indicates that one only uses OT cost as in equation (2.1), while $\alpha = 1$ indicates pure Gromov–Wasserstein OT that penalizes the change in global structure and is less fine-grained. We show that Gromov–Wasserstein appears to smooth out the velocity field too much while OT can be too restrictive, hence a linear combination of both ($\alpha = 0.5$) can yield a smoother velocity field compared to $\alpha = 0$ or $\alpha = 1$.

bioRxiv preprint doi: https://doi.org/10.1101/2024.09.12.612590; this version posted September 16, 2024. The copyright holder for this preprint (which was not certified by peer review) is the author/funder, who has granted bioRxiv a license to display the preprint in perpetuity. It is made available under aCC-BY 4.0 International license.

S7 Fig. True network and results from different approaches, illustrated on one instance of FN8 dataset simulated by HARISSA. Top: resulting graphs from different approaches. Bottom: weight matrices used to construct the graphs. The correlation approach has default parameter $(\alpha, \epsilon) = (0.5, 0.01)$, while the regression approaches all have $\lambda = 1$, and $r = 0, 0.5, 1.0$ respectively. While the correlation was able to capture most of the structure, the two approaches with l_1 regression were able to further reduce the density of graph.

S8 Fig. Related to Figure 6. Results of HARISSA, CARDAMOM, GENIE3, and SINCERITIES on scGEM dataset [6], with identical layout as Figure 6. GENIE3 does not identify the type of regulation and all edges are visualized in blue.

S9 Fig. Related to Figure 8. First two principal components of mouse data according to time and the velocity field identified by optimal transport.

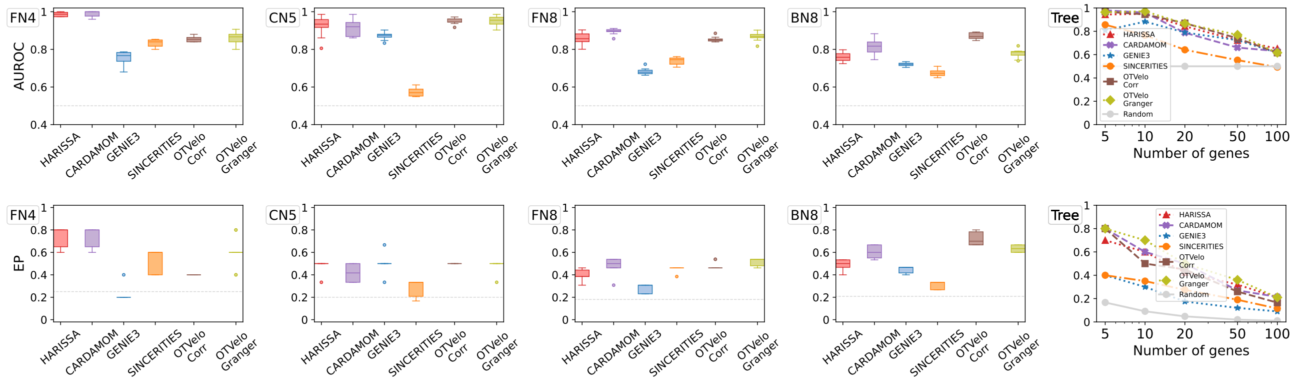

Figure S1: *Other performance metrics except AUPRC on datasets simulated from HARISSA [34], including Area under Receiver Operating Characteristic (ROC) curves and early precision as defined by [22].*

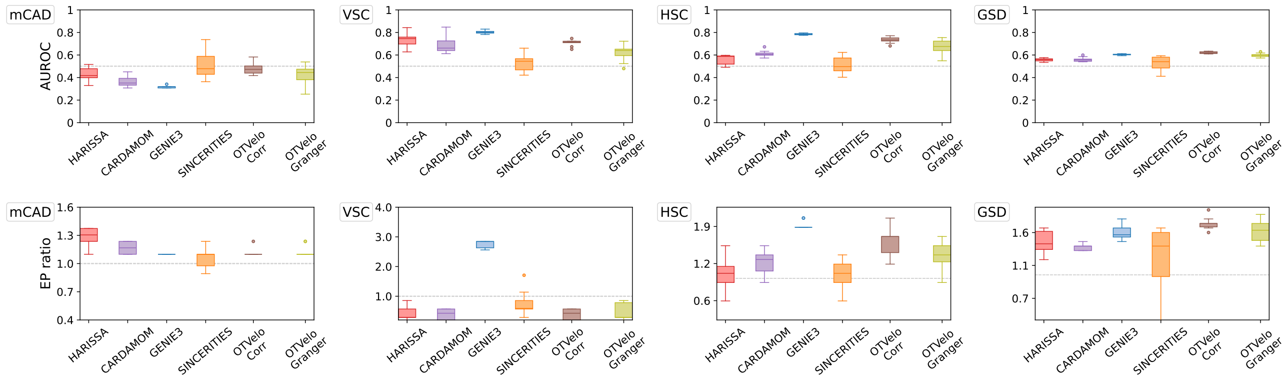

Figure S2: *AUROC and EP ratio on datasets simulated from curated networks and BoolODE [22]. The EP ratio equals the early precision value divided by the random baseline, and the value should be greater than one if the performance is better than a random classifier.*

bioRxiv preprint doi: https://doi.org/10.1101/2024.09.12.612590; this version posted September 16, 2024. The copyright holder for this preprint (which was not certified by peer review) is the author/funder, who has granted bioRxiv a license to display the preprint in perpetuity. It is made available under aCC-BY 4.0 International license.

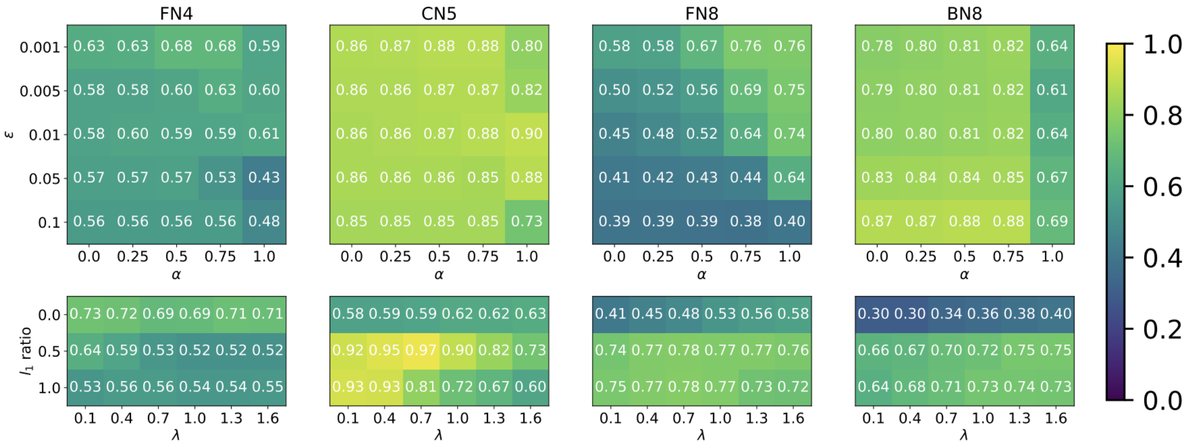

Figure S3: *Dependence of median AUPRC on hyperparameters (1)* (α, ϵ) *for OTVelo-Corr, and (2)*(λ, r) *for OTVelo-Granger on HARISSA simulations. Brighter is better.*

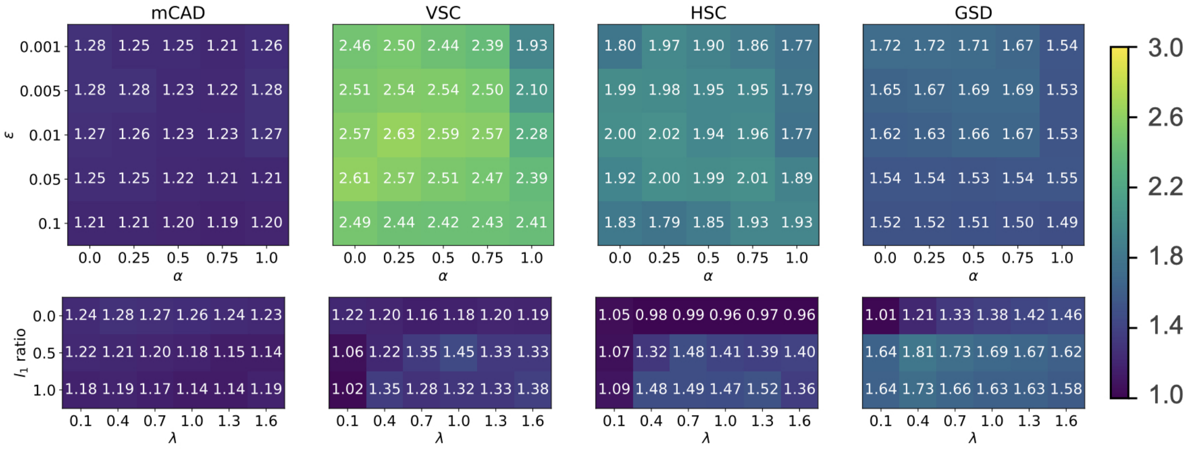

Figure S4: *Dependence of median AUPRC-based metrics on hyperparameters (1)* (α, ϵ) *for OTVelo-Corr, and (2)*(λ, r) *for OTVelo-Granger on Curated models. Brighter color is better.*

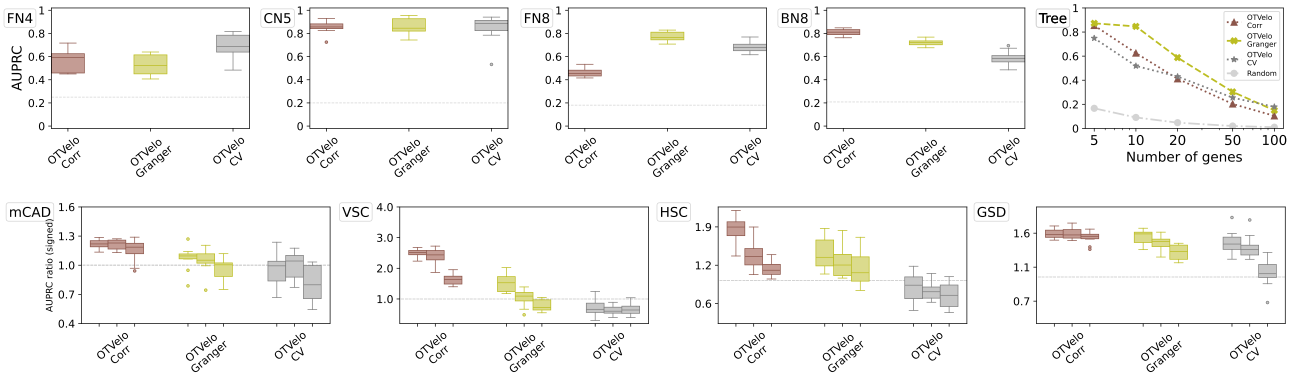

Figure S5: *AUPRC given signed prediction when cross validation is performed to find* (r, λ)*. The approach with default parameters* $r = 0.5, \lambda = 1$ *is labeled 'OTVelo-Granger', and 'OTVelo-CV' indicates a strategy of picking* (r, λ) *via a 5-fold cross validation over a grid* $r \in \{0, 0.5, 1.0\}$ *and* $\lambda \in \{0.1, 0.4, 0.7, 1.0, 1.3, 1.6\}$*, the same grid as in Figures S3 and S4.*

bioRxiv preprint doi: https://doi.org/10.1101/2024.09.12.612590; this version posted September 16, 2024. The copyright holder for this preprint (which was not certified by peer review) is the author/funder, who has granted bioRxiv a license to display the preprint in perpetuity. It is made available under aCC-BY 4.0 International license.

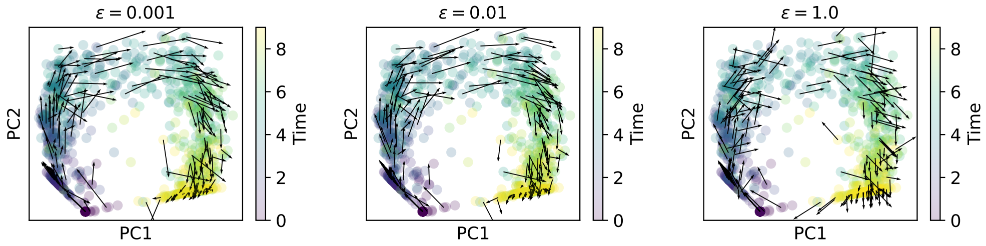

(a) *Varying* ϵ *with fixed* $\alpha = 0.5$

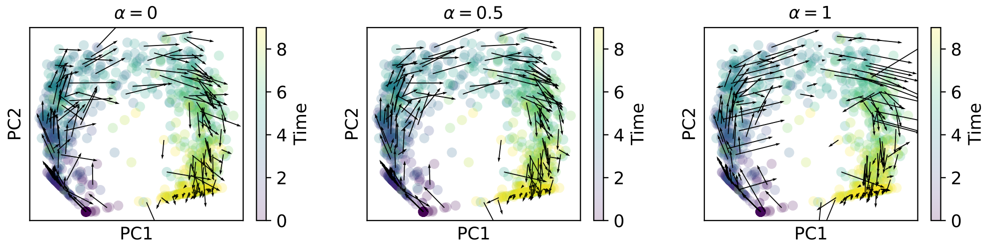

(b) *Varying* α *with fixed* ϵ*=0.01*

Figure S6: *Dependence of velocity field on* (α, ϵ) *illustrated on one instance of FN8 dataset simulated by HARISSA. Top: velocity field with* $\alpha = 0.5$ *and different* ϵ*. Lower values of* ϵ *gives velocity fields of good smoothness but can take significant computational time or even fail to converge when* $\epsilon < 0.001$*, while bigger value of* ϵ *results in non-smooth velocity field. Bottom: velocity field with fixed* $\epsilon = 0.01$ *but different* α*.* $\alpha = 0$ *indicates that one only uses OT cost as in equation* (2.1)*, while* $\alpha = 1$ *indicates pure Gromov–Wasserstein OT that penalizes the change in global structure and is less fine-grained. We show that Gromov–Wasserstein appears to smooth out the velocity field too much while OT can be too restrictive, hence a linear combination of both (*$\alpha = 0.5$*) can yield a smoother velocity field compared to* $\alpha = 0$ *or* $\alpha = 1$*.*

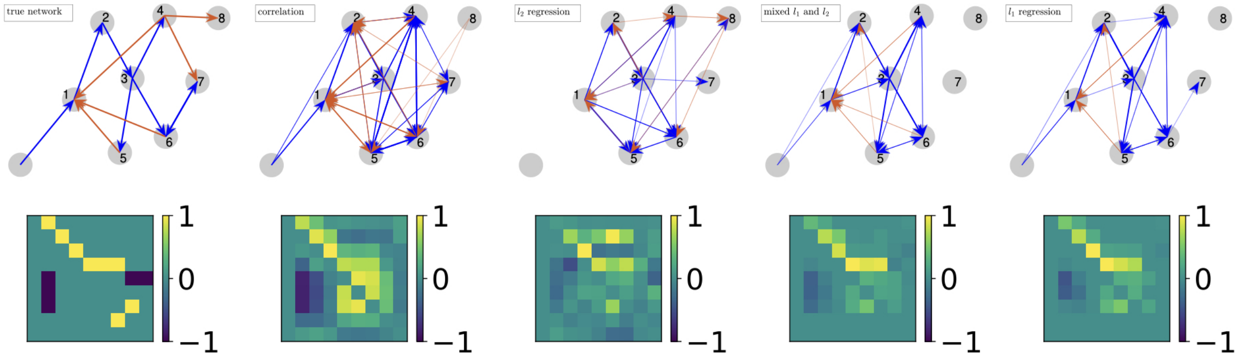

Figure S7: *True network and results from different approaches, illustrated on one instance of FN8 dataset simulated by HARISSA. Top: resulting graphs from different approaches. Bottom: weight matrices used to construct the graphs. The correlation approach has default parameter* $(\alpha, \epsilon) = (0.5, 0.01)$*, while the regression approaches all have* $\lambda = 1$*, and* $r = 0, 0.5, 1.0$ *respectively. While the correlation was able to capture most of the structure, the two approaches with* l_1 *regression were able to further reduce the density of graph.*

bioRxiv preprint doi: https://doi.org/10.1101/2024.09.12.612590; this version posted September 16, 2024. The copyright holder for this preprint (which was not certified by peer review) is the author/funder, who has granted bioRxiv a license to display the preprint in perpetuity. It is made available under aCC-BY 4.0 International license.

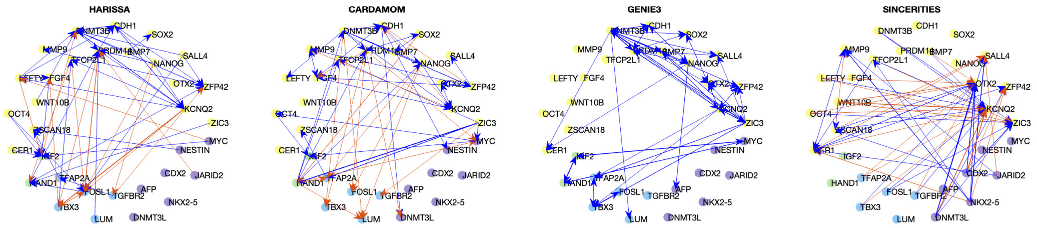

Figure S8: *Results of HARISSA, CARDAMOM, GENIE3, and SINCERITIES on scGEM dataset [6], with identical layout as Figure 6. GENIE3 does not identify the type of regulation and all edges are visualized in blue.*

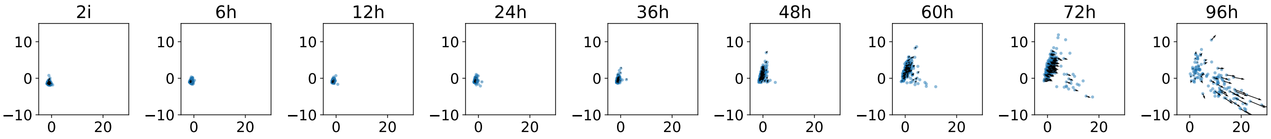

Figure S9: *First two principal components of mouse data according to time and the velocity field identified by optimal transport.*
